# Probiotics supplementation and insulin resistance: a systematic review

**DOI:** 10.1186/s13098-020-00603-6

**Published:** 2020-11-11

**Authors:** Bárbara Izabel Moraes Salles, Débora Cioffi, Sandra Roberta G. Ferreira

**Affiliations:** Departamento de Epidemiologia, Faculdade de Saúde Pública, School of Public Health, University of Sao Paulo, Av. Dr. Arnaldo, 715, São Paulo, SP CEP 01246-904 Brazil

**Keywords:** Probiotics, Gut microbiota, Endotoxemia, Insulin resistance, Type 2 diabetes mellitus, Prediabetes, Inflammation, Short-chain fatty acids

## Abstract

**Background:**

Research on intestinal microbiota has grown considerably, as well as the interest on probiotics’ supplementation effects on metabolism. Considering high prevalence rates of metabolic diseases linked by insulin resistance, we performed a systematic review of existing literature which addressed the role of probiotics in modulating insulin sensitivity in animals and humans.

**Methods:**

This systematic review was based on PRISMA guidelines. Searches for original articles published in English from 1990 to January 2020 were made in the electronic database of PubMed from the National Library of Medicine, using Medical Subject Headings to identify longitudinal studies conducted in animals and humans which reported effects of probiotics in a variety of insulin resistance parameters.

**Results:**

Overall, results from 27 probiotic interventions (*Lactobacillus*, *Bifidobacterium*, *Clostridium* and *Akkermansia*) indicated significant beneficial changes in insulin resistance measures in animal studies. Additionally, they improved lipid profile, inflammatory and oxidative markers, short-chain fatty acids production and microbiota composition. In seven clinical trials, samples and designs were heterogeneous. Five showed benefits in insulin resistance parameters and in two others no effect was detected.

**Conclusion:**

Available data regarding the effects of certain probiotics do not guarantee sustained amelioration of insulin resistance in humans. Consistent beneficial results for intestinal barrier function, immune system and metabolism were reported in animals may encourage long-term randomized clinical trials in people with obesity and cardiometabolic risk. Whether supplementation with probiotics in combination with medications and/or prebiotics, associated with a healthy lifestyle, will prove useful to attenuate insulin resistance requires further investigation.

## Introduction

Growing rates of obesity represent a global lifestyle-related health problem [1]. Its presence favors comorbidities and increases mortality mainly due to cardiometabolic complications [1]. In particular, visceral adipose tissue depot produces inflammatory cytokines inducing insulin resistance, involved in the pathophysiology of type 2 diabetes mellitus (DM), hypertension and dyslipidemia; this condition increases the risk of cardiovascular disease, the leading cause of death in most populations [2]. DM is also among the top ten causes of death; it is estimated that 463 million adults have DM and 374 million prediabetes worldwide and the number of diabetic people is predicted to increase to 700 million in 2045 [3]. This scenario has justified the search for underlying mechanisms of these lifestyle-related diseases.

Studies in animals and humans have emphasized the role of gut microbiota as a mediator of cardiometabolic disorders [4–6]. Trillions of microorganisms inhabit the gastrointestinal tract, exceeding the number of human cells by ten times [6]. They play a crucial role in immunity, nutrients’ absorption, bioactive molecules actions and in metabolism homeostasis [4, 7]. Diversity and stability of the microbiota are affected by genetic and environmental factors. An imbalance of bacterial communities is called dysbiosis that has been described in obesity and other states of insulin resistance [8].

Animal models provided initial evidence of differences in microbiota composition according to body adiposity. Predominance of the phylum *Firmicutes* was reported in obese mice [9]. Transplantation of fecal microbiota from lean donors to obese animals revealed the ability of bacterial content in changing body adiposity and related disorders in recipients [9–11]. In obese humans, a higher proportion of *Firmicutes* relative to *Bacteroidetes* was also described, although heterogeneous findings were further reported [4, 9]. Promising results of fecal transplantation have also emerged from clinical trials including obese individuals with metabolic disturbances [9].

Beyond the taxonomic classification, some authors have proposed describing clusters of bacterial genera named enterotypes [12]. Western diet, rich in protein and saturated fatty acids, was associated with the Bacteroides enterotype, while diet rich in complex carbohydrates and vegetables with the Prevotella enterotype [13].

Diets enriched with saturated fatty acids have been associated with increased abundance of gram-negative bacteria containing lipopolysaccharides (LPS) on their surface. This condition augments intestinal permeability and LPS translocation, leading to metabolic endotoxemia, inflammation and insulin signaling deterioration [5, 6, 9, 14]. On the other hand, fiber-rich diets have been associated with beneficial effects on energy balance [15]. Short-chain fatty acids (SCFA) derived from fiber fermentation acting on enteroendocrine cells resulting in local and remote benefits [16]. Gut-derived butyrate, acetate and propionate play a role in central regulation of satiety and metabolism and have been considered a relevant pathophysiological link between gut microbiota and disturbances of glucose metabolism [15]. In individuals with and without type 2 DM, comparisons of microbiota composition have shown lower abundance of a butyrate-producing bacterium and *Akkermansia muciniphila,* in diabetics [9, 14], while in mice dietary supplementation with butyrate ameliorated inflammation and insulin resistance [16].

Evidence on the potential of gut microbiota to improve inflammation and metabolic disturbances in diabetic mice [16] raised the hypothesis that probiotics could represent a therapeutic tool to attenuate microbiota-related mechanisms that generate insulin resistance in human diseases [5, 6]. Probiotics are preparations of microbial cells or their components that confer health benefits to the host when administered in adequate amounts. They are generally safe for human consumption and act through the microbiota modulation. Among potential benefits, those on improving glucose metabolism have been widely studied [17]. Considering the deleterious effects of Western diet in intestinal permeability to endotoxins [9], probiotics have been proposed to improve in microbiota composition. Evidence has indicated that intestinal barrier can be restored by increasing tight-junction proteins expression which reduces endotoxemia and ameliorates insulin sensitivity.

Based on results obtained from animal models and preliminarily in humans [18], probiotic interventions have been tested as a potential strategy for the prevention or treatment of type 2 DM. Several bacterial strains have been proposed to enhance abundance of bacteria which mitigate inflammation and insulin resistance, thus protecting against cardiometabolic diseases. Most studied interventions were based on *Lactobacillus*, *Bifidobacterium* and *Akkermansia muciniphila* [6, 9]. *Akkermansia* genus has the ability to breakdown mucins from the mucus layer providing oligosaccharides sources to be metabolized by resident microbiota.

Animal studies have demonstrated that obesity and DM induced by high-fat diet (HFD) is at least partially mediated by the gut microbiota. Supplementation of *Bifidobacterium breve* promoted less weight and visceral adipose tissue gains and improved lipid and glucose metabolism [19], while *Bifidobacterium animalis* was able to reverse the HFD-induced DM [20]. Animal studies with probiotics addressed to obesity-related outcomes have employed mainly *Lactobacillus* and *Bifidobacterium* species based on heterogeneous research protocols [21]. Experiments with *Lactobacillus* and *Bifidobacterium* mixture administered in humans resulted in improvement of insulin sensitivity [17, 22]. As a proof of concept, a small study in humans revealed encouraging results by the administration of live or pasteurized *A. muciniphila* [23].

The predominance of studies regarding probiotics effects on metabolism were conducted in animals. Supplementations were made with diverse bacteria in different amounts and different durations limiting comparison of their potential benefits. Experimental protocols also differed regarding the type of culture, formulations and storage conditions that could generate responses [4–6]. Protective cardiometabolic effects of probiotics in humans with or at-risk for cardiometabolic diseases were less investigated [5, 9, 24]. As far as we know, no systematic review in this issue included experiments conducted in animals as well as in humans.

Considering the burden of cardiometabolic diseases, the potentialities of probiotics supplementation as adjuvant tools for their control and the heterogeneity of the reported studies, a systematic review could indicate important gaps in this knowledge and orient further research. Although changes in gut microbiota composition by probiotics seem an attempt to minimize inflammation and insulin resistance, it is unclear whether they could attenuate such underlying mechanisms of lifestyle-related diseases highly prevalent in humans. Since modern dietary habits are determinants of metabolic disturbances, interventions on diet using probiotics’ supplementation represent an opportunity for prevention of insulin resistance-linked diseases. We performed a systematic review of existing literature in English which addressed the role of probiotics in modulating insulin sensitivity in animals and humans.

## Methods

This review was performed based on the PRISMA guidelines (Preferred Reporting Items for Systematic Reviews and Meta-Analysis). Detailed information is provided in Additional file 1.

### Search strategy

Searches for original research articles offered online, published in English from 1990 to January 2020, were conducted in the electronic database of PubMed from the National Library of Medicine. The following Medical Subject Headings (MeSH) were used in several combinations: “probiotics”, “lipopolysaccharides”, “gastrointestinal microbiome”, “intestinal mucosa”, “endotoxemia”, “fatty acids, volatile”, “lactobacillus”, “propionibacterium”, “bifidobacterium”, “saccharomyces”, “insulin resistance”, “insulin sensitivity”, “ type 2 diabetes”, “type II diabetes”, “prediabetes” and “glucose intolerance”. The terms “akkermansia” and “enterotype” are not MeSH descriptors although they were also searched considering their importance for this review’s purpose. Reference lists of included studies were additional sources.

### Eligibility criteria

Inclusion criteria were all longitudinal controlled studies with probiotics supplementing diet of animal models or humans and an available objective measurement of insulin sensitivity or resistance. Exclusion criteria were participants with type 1 DM, pregnant women, lack of information regarding the intervention with probiotics and review studies.

### Data handling

Two independent reviewers (BIMS, DC) assessed the titles and abstracts of all retrieved references to identify studies that potentially met eligibility criteria and eligible articles were retrieved in full text. If duplication of the same study was found, its data were included just once. Disagreements were solved through discussion with another reviewer (SRGF). All relevant study data were extracted into a Microsoft Excel database. Variables extracted included the following: year of publication, country of research, sample characteristics (type and size), experimental conditions (study design and protocol), probiotic intervention (strain type, doses, duration), primary parameter of insulin sensitivity/resistance (baseline and post-intervention) and secondary parameters. Double entry was used to assure the accuracy of data included in the database. These data were summarized in tables, for animal models and humans separately.

**Fig. 1:**
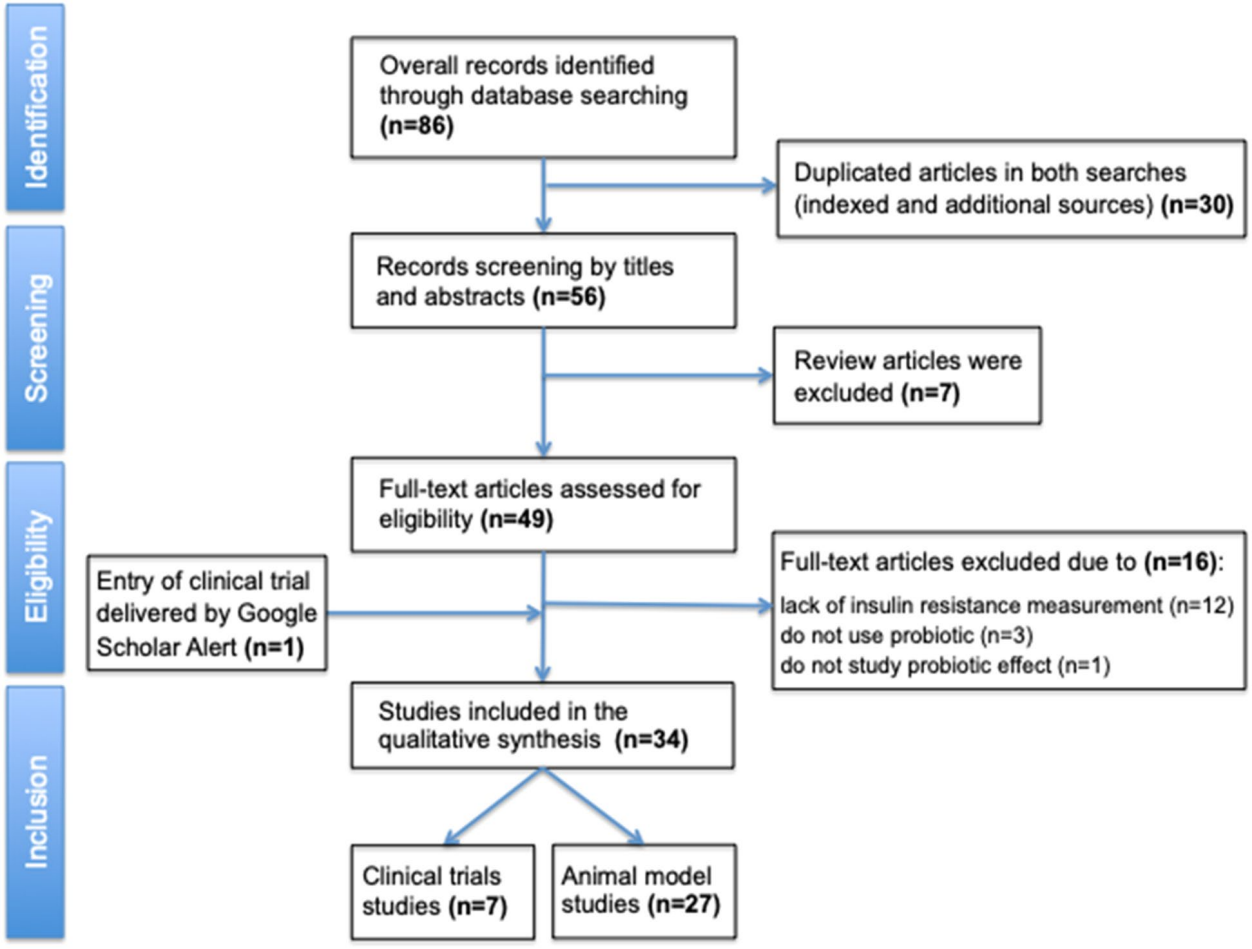
Flowchart of articles selection for this systematic review based on PRISMA

The flowchart of articles selected are shown in Fig. 1. Sum of MeSH indexed search and free search on PubMed database returned 86 articles; 30 appeared in duplicate and were excluded, resulting in 56 articles for this review. Initial screening of abstracts was made in 56 articles conducted in animals and humans. Among those, seven review articles were excluded. A total of 49 studies had full text reading by two reviewers (BIMS; DC) and from these, 16 were excluded due to lack of an objective measure of insulin resistance; not being a probiotic; and not analyzing the effect of the probiotic, resulting in 33 studies included in our systematic review. Further, one recent article [23] that was not detected using the defined MeSH but was automatically delivered (Google Scholar Alerts) to one author (SRGF) was also included.

A total of 34 articles were analyzed, distributed in 27 studies involving animal models and seven clinical trials. Probiotics used in the interventions (mainly *Lactobacillus* and *Bifidobacterium*) have already recognized benefits on health. Most studies (82%) were carried out in the last 5 years in different continents. Studies’ origin was concentrated in Asia (China, Japan, Korea and India), Europe (France, Spain, Sweden and Austria) and one country in Latin America (Brazil).

The totality of animal studies included in this review was conducted in rodents. In the majority of these experiments HFD was used to induce obesity and insulin resistance and different probiotics were chosen to evaluate their performance on several metabolic parameters. One study [25] used high-fructose diet to induce obesity and glucose intolerance and another maintained a normal chow diet [26]. In clinical trials, no special dietary recommendation was made. All trials were randomized and controlled, being six double-blinded and one single-blinded, four were multicentric and three were single-center. Sample characteristics were diverse, having included healthy participants as well as individuals with type 2 DM or metabolic syndrome (MS).

### Analysis and interpretation

Main details of the studies selected including probiotic strains, doses and other experimental characteristics, effects of probiotic interventions on parameters of glucose metabolism and other relevant outcomes are shown in Tables 1 and 2.

Reviewer risk of bias was minimal due to strict collection of eligible articles from literature and because only statistically significant findings were considered to interpret the study result by three investigators. The only exception and possible source of bias could be related to inclusion of a recent small clinical trial with *A. muciniphila* by Depommier et al. [23]. Despite its preliminary nature, authors agreed about the relevance of this inclusion based on promising results for clinical practice. There was always a consensus among authors in relation to interpretation of the effects of all interventions in parameters of insulin sensitivity or resistance provided in the studies analyzed.

## Results

### Animal studies

Main results related to glucose metabolism of 27 animal studies are shown in Table 1 and additional findings are mentioned in the text. The majority of them was performed using strains of *Lactobacillus* (n = 20) and *Bifidobacterium* (n = 4), as single-strain probiotics or blends of multiple strains (*Lactobacillus* + *Bifidobacterium*). Two studies used *Akkermansia* and only one used *Clostridium*. Few studies reported findings regarding microbiota, presenting proportions of bacteria belonging to the main phyla; sometimes, the ratio was mentioned as *Firmicutes*-*to*-*Bacteroidetes* and others *Bacteroidetes*-*to*-*Firmicutes*. In four articles, effects of probiotics were compared to oral hypoglycemic drugs (metformin, pioglitazone and vildagliptin).

**Table 1:** Description of the included animal studies

Reference	Research protocol (duration and experimental groups)	Probiotic type and dose	Insulin resistance parameters	Effects on glucose metabolism	Other significant outcomes
Andersson et al. [18] 2009, Sweden	18 weeks Groups: Control (HFD diabetic control) Treated Lp DSM 15313 (HFD + probiotic)	*Lactobacillus plantarum DSM 15313* 2.4 × 10^9 CFU	Plasma insulin in OGTT and IVGTT	At the end of the intervention, insulin levels in response to OGTT (p < 0.05)—but not to IVGTT—were lower in the treated group than the control group.	Body weight increase in the probiotic group seemed to be due to lean mass gain rather than fat mass. IL-6 was below detection limit and signs of systemic inflammation were not detected. No difference in total cholesterol, non-esterified free fatty acids and adiponectin levels were found.
Kondo et al. [19] 2010, Japan	8 weeks Groups: Control (HFD diabetic control) B-3L (HFD + *B. breve* B-3L) B-3H (HFD + *B. breve* B-3H)	*Bifidobacterium breve B*-*3L* 10^8 CFU or *Bifidobacterium breve B*-*3H* 10^9 CFU	Plasma insulin and HOMA-IR	Reductions in glucose, insulin and HOMA-IR, plasma glucose were found only in treated groups (p < 0.05) being more pronounced in B. breve B-3H than B-3L.	Supplemented groups had lower mean body and epididymal fat weight. Expressions of adiponectin and proglucagon genes were higher in both probiotic groups; fasting-induced adipose factor expression was higher only in the B-3H group. No change was observed in *Bacteroidetes*-*to*-*Firmicutes* ratio.
Amar et al. [20] 2011, France	6 weeks 8 Experiments: Exp.1, 4, 5: control; HFD-fed for 1 wk; HFD-induced diabetic 4 weeks Exp.2: WT control, HFD-fed for 4 weeks, Nod1 −/− NC, Nod1 −/− 4 weeks WT control, HFD-fed for 4 weeks, Nod2 −/− NC, Nod2 −/− 4 weeks WT, ob/ob, ob/ob CD14−/−, CD14 −/− Exp.3: WT control, Myd88 −/− Exp.6, 7: *L. lactis, L. lactis leptin* Exp.8: NC, HFD + vehicle, HFD + *B420*	*Bifidobacterium animalis subsp. lactis 420* 10^9 CFU	Fasting insulin and AUC after OGTT and IPGTT	In OGTT, Nod2 knockout mice had similar glycemia to HFD-fed WT. Glycemic values of WT HFD-fed were significantly higher than other knockout groups for Nod1 and WT controls. In IPGTT, ob/ob mice had the highest glycemic level compared to ob/ob cd14−/−, WT and cd14 −/− groups (p < 0.05), and these 3 had similar values. In IPGTT, comparing knockouts for myeloid differentiation protein (Myd88) and WT, fasting glucose, insulin and insulin resistance in the Myd88 knockout were increased compared to WT. Glucose intolerance was slightly improved and fasting insulin remained unchanged when comparing to L. lactis and *L. lactis* leptin groups. Fasting glucose and insulin fell after *L. lactis* intervention (p < 0.05).	Lack of microbial pattern recognition receptors nucleotide-binding oligomerization domain-containing protein 1 (Nod1) or cluster of differentiation 14 (CD14) prevented intestinal bacteria translocation and inflammation, while Myd88 knockout and ob/ob mouse showed increased translocation. Probiotic treatment can reduce dysbiosis and regulate intestinal bacterial adherence, translocation and metabolism, resulting in improvement of inflammation and metabolic status.
Zhang et al. [25] 2013, China	9 weeks or 13 weeks Groups: Control LP (*L. casei*-Zhang preventive) HMI (hyperinsulinemia model-9 weeks) LT (*L. casei* Zhang -therapeutic) HMII (hyperinsulinemia model-13 weeks)	*Lactobacillus casei Zhang* 10^9 CFU	Insulin AUC after OGTT	Fructose-treated group developed impaired glucose tolerance. Compared to HMI group, the LP had a non-significant decrease in the glucose peak, while LT reduced the glycemic peak compared to the HMII group (p = 0.048). During OGTT, the LP group prevented worsening of glucose intolerance, with improvement in glucose tolerance at HMI and in the LT group (p < 0.05).	Probiotic-treated group increased osteocalcin level and improved several markers of liver function and gene expressions (bile acids secretion, decreased malonaldehyde levels and upregulation of liver X receptor alpha, peroxisome proliferator-activated receptor and adiponectin receptor 2 gene expression).
Toral et al. [28] 2014, Spain	12 weeks-Exp.1: Control (ND control) Control-treated (ND + probiotic) Obese (HFD diabetic control) Obese-treated (HFD + probiotic) 2 weeks-Exp.2: Control (ND control) Control-treated (ND + probiotic)	*Lactobacillus coryniformis CECT5711* 10^8 CFU	Fasting insulin, HOMA-IR and AUC after OGTT	AUC after an OGTT (p < 0.01) and HOMA-IR (p < 0.05) were worse in mice under HFD than in controls. Non-treated obese group had higher AUC (p < 0.01) than the probiotic-treated obese group, whose performance was similar to control groups. Probiotic treatment reduced basal glycemia and insulin resistance, and improved glucose tolerance without significant changes in insulinemia.	Treatment induced changes in microbiota composition causing an improvement in gut barrier and metabolic endotoxemia (reduced LPS levels). Also, the decreased TNF-α expression in liver indicated an improved inflammatory status. An endothelial-protective effect was suggested by reversing the endothelial dysfunction related nitric oxide-dependent vasodilatation and restoring the increased vessel superoxide levels by reducing the NADPH oxidase activity and increasing antioxidant enzymes.
Alard et al. [42] 2016, France	7 weeks Groups: LFD (ND control) LFD + PBS (ND + multistrain) HFD (HFD diabetic control) HFD + PBS (HFD + multistrain)	*Multi*-*strain mixture:* *Lactobacillus rhamnosus LMG S*-*28148* *Bifidobacterium lactis LMG P*-*28149* 5 × 10^8 *CFU of each strain*	Fasting insulin, HOMA-IR and AUC after IPGTT	AUC after IPGTT indicated that HFD-fed animals treated with a multi-strain probiotic mixture reduced fasting glucose and insulin levels (p < 0.05) in contrast to those not treated with probiotics. HOMA-IR was lower in the HFD-mix group compared to the control group (p < 0.05), suggesting greater insulin sensitivity that was confirmed by the IPGTT.	Probiotic mixture remodelled immune cells in adipose tissue and improved adiposity and dyslipidemia. Microbiota composition (increased *A. muciniphila* and *Rikenellaceae* and decreased *Lactobacillaceae* abundances) was changed, expression of the SFCA receptor was restored, ameliorating intestinal uptake of fatty acids. In in vitro simulation of luminal and mucosal environment, the probiotic mixture favored butyrate and propionate production.
Hsieh et al. [29] 2016, Taiwan	12 weeks Groups: Control (ND control) Control + HK Lr263 (ND + *L. reuteri* Lr263 HK) Control + Lr263 (ND + *L. reuteri* Lr263 live) HFD (HFD diabetic control) HFD + HK Lr263 (HFD + *L. reuteri* Lr263 HK) HFD + live Lr263 (HFD + *L. reuteri* Lr263 live)	*Lactobacillus reuteri GMNL*-*263 (HK Lr263)* *Heat*-*killed/Lyophilized* 2 × 10^9 CFU *Lactobacillus reuteri GMNL*-*263 (Lr263)* *Live* 2 × 10^9 CFU	Fasting insulin, HOMA-IR and AUC after OGTT	HOMA-IR was higher in HFD group than in control one but reduced after treatments with lyophilized or live probiotics (p < 0.05). It did not differ between the control groups. Glycemic values in OGTT revealed that administration of live or lyophilized Lr263 attenuated HFD-induced glycemia increase. No differences in OGTTs of the 3 control groups, with or without probiotics, were observed. Increased insulin and glucose levels in HFD group were reduced by administration of live or lyophilized Lr263 (p < 0.05).	Live or lyofilized probiotics reduced weight gain and improved lipid profile. They suppressed the increased macrophage infiltration in adipose tissue and decreased proinflammatory-associated gene expressions in adipose and hepatic tissues contributing to attenuate hepatic steatosis. Treatment restored the proportion of probiotic bacteria and decreased pathogens improving intestinal barrier.
Lim et al. [31] 2016, Korea	4,5 weeks Groups: LFD (ND control) LFD-67 (ND + *L. sakei* OK67) HFD (HFD diabetic control) HFD-67 (HFD + *L. sakei* OK67)	*Lactobacillus sakei OK67* 10^9 CFU	Fasting insulin and AUC after OGTT	Probiotic treatment reduced plasma glucose and AUC after OGTT in animals under HFD (p < 0.05) but not in mice with LFD. Animals fed with HFD increased insulin levels and probiotic treatment reduced the increase in insulinemia (p < 0.05).	Probiotic reduced inflammation by decreasing TNF-α and IL-1β expressions and increasing IL-10 and tight junction protein expressions in the colon. Treatment inhibited NF-κB activation in LPS-stimulated peritoneal macrophages. Downregulated expression of peroxisome proliferator-activated receptor γ, fatty acid synthase, and TNF-α in adipose tissue were also observed.
Shang et al. [15] 2016, China	12 weeks Groups: ND (ND control) HFD (HFD diabetic control) HFD-Cb (HFD + probiotic)	*Clostridium butyricum CGMCC0313.1* 2 × 10^8 CFU	Fasting insulin and glucose tolerance during ITT and OGTT	The performance of OGTT and ITT for treated group revealed an improved glucose tolerance and relatively better insulin sensitivity with a reduced fasting insulinemia.	Treated group had lower weight gain, hepatic steatosis, accumulation of lipids in the liver. Also, lower TNF-α, IL-1B and MCP-1 levels, and higher IL-10 and IL-22 levels were found. SCFA production was restored, claudin-1 and occludin expressions were increased and LPS level decreased.
Balakumar et al. [33] 2016, India	24 weeks Groups: NPD (ND control) HFD (diabetic control) HFD + LGG (HFD + *L. rhamnosus* GG) HFD-MTCC 5690 (HFD + *L. rhamnosus* MTCC5690) HFD-MTCC 5689 (HFD + *L. rhamnosus* MTCC5689) HFD + metformin HFD + vildagliptin	*Lactobacillus rhamnosus GG* 1.5 × 10^9 CFU *Lactobacillus plantarum Lp91 (MTCC5690)* 1.5 × 10^9 CFU *Lactobacillus fermentum Lf1 (MTCC5689)* 1.5 × 10^9 CFU	Fasting insulin, HOMA-IR, AUC after OGTT and glucose tolerance during ITT	After 16 weeks, there was a significant improvement in insulin sensitivity (reduction in HOMA-IR, p < 0.05) in all treated groups but not in HFD control. Groups treated with LGG, MTCC5689, metformin or vildagliptin had reductions in glucose levels during OGTT (p < 0.05). All groups treated with drugs or probiotics improved the AUC after ITT but not HFD control group.	Probiotic interventions (especially MTCC5689) reduced lipids, normalized GLP-1 levels and restored endoplasmatic reticulum stress markers. Increased transcription for tight junction markers and decreased LPS levels were also observed. Except for LGG, the other 2 probiotic groups increased transcription for adiponectin in fat tissue. Transcriptions of inflammatory (TNF-α, IL-6 and MCP-1), lipogenesis, and gluconeogenesis gene expression in liver were reduced and normalized by probiotic intervention.
Zhao et al. [26] 2017, China	5 weeks Groups: NCD (ND control) NCD + P (ND probiotic)	*Akkermansia muciniphila* 2 × 10^8 CFU	Fasting insulin and AUC after IPGTT	Despite no change in fasting plasma glucose, supplementation with *A. muciniphila* to mice fed with a standard diet, IPGTT showed improvement in glucose tolerance (p < 0.01). Fasting insulinemia was comparable between the two groups.	Intervention induced reduction in body weight gain, fat mass and gene expressions related to fatty acid synthesis and transport in liver and muscle—alleviating endoplasmic reticulum stress; and in low-grade inflammation reflected by increased anti-inflammatory factors such as α-tocopherol and β-sitosterol and decreased levels of LPS-binding protein and leptin..
Aoki et al. [41] 2017, Japan	6 weeks Exp.1: NCD (ND control) HFD (HFD diabetic control) HFD BlaG (*B. lactis* GCL2505) Exp.2: HFD (HFD diabetic control) HFD BlaG (*B. lactis* GCL2505) HFD BloJ (*B. longum* JCM1217T)	*Bifidobacterium lactis GCL2505 (BlaG)* 10^9 CFU *Bifidobacterium longum JCM1217T (BloJ)* 10^9 CFU	Insulin and AUC after OGTT	Improvement in glucose tolerance assessed by OGTT in BlaG group (p < 0.05) when compared to diabetic control and BloJ groups. No significant difference was detected between the last 2 groups.	BlaG treatment improved metabolic disorders by modulating gut microbiota enhancing Bifidobacterium, reduced visceral fat accumulation and increased GLP-1 and SCFA levels (especially acetate). BloJ had no effect on these parameters.
Singh et al. [34] 2017, India	6 weeks Groups: Control (ND control) HFD + SM (HFD diabetic control) HFD + LGG (HFD + *L. rhamnosus* GG) HFD + 17 (HFD + *L. rhamnosus* NCDC17)	*Lactobacillus rhamnosus GG* 8 to 8.5 log CFU *Lactobacillus rhamnosus NCDC17* 9.5 to 10 log CFU	Fasting insulin and AUC after OGTT	Treated groups had significant reductions in fasting insulin and AUC (p < 0.05) compared to diabetic control group.	LC group had lower plasma glucose, glycated hemoglobin, leptin, TNF-α, IL-6, LDL-c and higher HDL-c levels. Probiotic treatment increased expressions of phosphatidylinositol-3-kinase and glycogen synthase genes and decreased of glycogen synthase kinase 3 beta mRNA. Pancreas histology showed a recovery of islets cells. Improved production of SCFA (mainly acetate and butyrate) and decreased *Firmicutes*-*to*-*Bacteroidetes* ratio were found.
Li et al. [32] 2017, China	8 weeks Groups: NC (ND control) DC (HFD diabetic control) P (HFD + pioglitazone) LC (HFD + *L. casei*)	*Lactobacillus casei CCFM419* 8 × 10^9 CFU	Fasting insulin, HOMA-IR, AUC after OGTT and glucose tolerance during ITT	Probiotic treated but not control animals showed decreases in HOMA-IR (p < 0.05), indicating improvement in insulin sensitivity in diabetic mice. LC group showed significant lower glucose AUC after OGTT, suggesting that the probiotic could delay the onset of hyperglycemia and ameliorate the impaired glucose tolerance. Probiotic and pioglitazone brought similar results.	LC group had lower fasting and postprandial glucose, glycated hemoglobin, leptin, TNF-α, IL-6 and LDL-c levels and higher HDL-c. Probiotic treatment increased expressions of phosphatidylinositol-3-kinase and glycogen synthase genes and decreased of glycogen synthase kinase 3 beta mRNA. Pancreas histology showed a recovery of islets cells. Also, decreased *Firmicutes*-*to*-*Bacteroidetes* and improved production of SCFA (acetate and butyrate) were found.
Wang et al. [16] 2017, China	12 weeks Groups: C (ND control) M (HFD diabetic control) P (HFD + pioglitazone) 10^8 (HFD + *L. casei* 10^8) 10^9 (HFD + *L. casei* 10^9) 10^10 (HFD + *L. casei* 10^10)	*Lactobacillus casei CCFM419* 10^8 or 10^9 or 10^10 CFU	Fasting insulin, HOMA-IR and glucose AUC during OGTT	Teatment induced lower glucose AUC, insulin level and HOMA-IR (p < 0.05). Probiotic doses of 10^8 and 10^9 CFU showed better ability of improving insulin resistance than 10^10 CFU.	Probiotic groups had lower TNF-α levels and protective effects in islet cells. Acetate, butyrate and GLP-1 levels were increased (at 10^9 and 10^10 CFU doses) and *Firmicutes*-*to*-*Bacteroidetes* ratio reduced. All doses of probiotic had some improvement in lipid profile, but only the 10^9 CFU dose had significant reduction in triglycerides, total cholesterol and LDL-c. The same dose recovered significantly stress oxidative markers (SOD, glutationa, GPx and MDA) and decreased IL-6.
Bagarolli et al. [43] 2017, Brazil	5 weeks Groups: C (ND control) CPB (ND + probiotic pool) DIOPF (HFD diabetic control) DIOPB (HFD + probiotic pool)	*Mix of Lactobacillus rhamnosus,* *Lactobacillus acidophilus,* *Bifidobacterium bifidumi* 6 × 10^8 CFU *each*	Insulin and glucose AUC after an IPGTT and glucose infusion rate during hyperinsulinemic euglycemic clamp	Submitted to IPGTT, DIOPB (HFD + probiotics) animals had better glucose and insulin profiles than DIOPF (HFD control). DIOPF group exhibited a greater reduction in glucose infusion rate compared to control animals. Also, probiotic treatment partially restored insulin sensitivity in DIOPF. Probiotic administration did not change any parameter of normal-fed animals.	Probiotics recovered intestinal permeability, LPS translocation, systemic low-grade inflammation and induced an improvement in hypothalamic insulin and leptin resistance.
Zhang et al. [27] 2018, China	4 weeks Groups: NC (ND control) NH (normal high-dose—5 × 10^8 live *A. muciniphila*) DC (HFD diabetic control) DH (diabetic high-dose-5 × 10^8 live *A. muciniphila*) DL (diabetic low-dose-5 × 10^6 live *A. muciniphila*) DP (diabetic-5 × 10^8 pasteurized *A. muciniphila*) DM (HFD diabetic metformin)	*Akkermansia muciniphila* 5 × 10^8 live CFU or 5 × 10^6 live CFU or 5 × 10^8 pasteurized CFU	Insulin and glucose AUC after OGTT	NC groups showed milder fluctuations and lower glycemic peak than HFD groups (p < 0.001). No significant difference in insulin and glucose values was observed between the HFD probiotic groups during OGTT; only metformin group had a lower glycemic peak than control (p < 0.05).	All diabetic groups treated had significantly lower hepatic glycogen and TNF-α levels, while DL, DP and DM groups also had significantly lower levels of LPS, MDA, plasminogen activator inhibitor-1 and GLP-1. DC, DP and DM groups had significantly higher triglycerides levels, DM group higher total cholesterol levels, and all diabetic treated groups had significantly higher HDL-c levels.
Lee et al. [7] 2018, Korea	5 weeks Groups: control (ND control) HFD (HFD diabetic control) HFD + Ln4 (HFD diabetic + *L. plantarum* Ln4)	*Lactobacillus plantarum Ln4* 5 × 10^8 CFU	Fasting insulin, HOMA-IR, AUC after OGTT and glucose tolerance during ITT	Insulin levels decreased significantly in groups treated with probiotic which induced a 40.6% reduction of the increased HOMA-IR value induced by HFD feeding. AUCs obtained from OGTT and ITT reduced significantly.	Ln4 administration strongly attenuated weight gain and reduced levels of triglycerides and adipokines, C-reactive protein, insulin-like growth factor binding protein-3, leptin, lipocalin-2 and MCP-1. Also, it regulated hepatic expression of insulin receptor substrate 2, serine/threonine-protein kinase and AMP-activated protein kinase and improved expression of lipoprotein lipase.
Kikuchi et al. [40] 2018, Japan	4 weeks Groups: Chow-Ct (ND control) Chow-400 (ND + probiotic 400 mg/kg dose) HFD-Ct (HFD diabetic control) HFD-200 (HFD + probiotic 200 mg/kg dose) HFD-400 (HFD + probiotic 400 mg/kg dose)	*Bifidobacterium longum BR*-*108 sterilized* 200 mg/kg or 400 mg/kg	Insulin AUC after OGTT and glucose AUC after ITT	AUCs obtained from OGTT and ITT showed improvement in insulin resistance and glucose tolerance (p < 0.05) in both, HFD-200 and HFD-400 groups after probiotic treatment. In other groups no significant difference was found.	Supplements of sterilized bacterial cells reduced weight gain, epididymal body fat mass, cholesterol and triglycerides levels, circulating lipopolysaccharides and liver triglycerides and had beneficial effects in intestinal microbiota.
Natividad et al. [21] 2018, France	4 weeks Groups: CD (ND control) HFD (HFD diabetic control—low *B.wadsworthia*) HFD Bw + (HFD + *B. wadsworthia*) HFD Bw + Lr + (HFD + *B. wadsworthia* + *L. rhamnosus*)	*Lactobacillus rhamnosus CNCM I*-*3690* 10^9 CFU	Fasting insulin, HOMA-IR and AUC after OGTT	Probiotic induced significant reductions in fasting plasma glucose, insulin and HOMA-IR. AUC obtained by OGTT further revealed that HFD Bw + Lr (mice with abundance of *B. wadsworthia* and treated with probiotic) tended (p = 0.058) to show a better glycemic control than HFD Bw + .	*B. wadsworthia* synergizes with HFD worsening systemic and mucosal inflammation. The probiotic intervention showed that it can be at least partly reversed by *L. rhamnosus*, which was associated with increased butyrate and propionate levels.
Niibo et al. [35] 2018, Japan	3 weeks Groups: Control (HFD diabetic control) LG2055 (HFD + probiotic)	*Lactobacillus gasseri SBT 2055 (LG2055)* 6 × 10^7 CFU	Fasting insulin and in response to OGTT	Both, fasting insulin and in response to OGTT, did not differ between groups. However, in diabetic animals, probiotic administration improved insulin secretion by attenuating inflammation.	Probiotic intervention increased HDL-c, C-peptide, liver glycogen and total SCFA content and decreased inflammatory cytokine levels. Also, higher expressions of insulin genes and insulin promoter factor 1 were found.
Thiennimitr et al. [8] 2018, Thailand	12 weeks Groups: ND (ND control) HFD (diabetic control) HFD *L.paracasei* (HFD + probiotic) HFD XOS (HFD + prebiotic) HFD HII01 + XOS (HFD + synbiotic)	*Lactobacillus paracasei HII01* 10^8 CFU	Fasting insulin, HOMA-IR and AUC after OGTT	Groups supplemented with probiotic improved insulin sensitivity as indicated by decreased insulin, HOMA-IR and plasma glucose AUC (p < 0.05).	Probiotic treated group improved lipid profile, attenuated dyslipidemia as indicated by decreased total and LDL-cholesterol levels. Also, a decrease in *Firmicutes*-*to*-*Bacteroidetes* ratio was observed.
Chunchai et al. [37] 2018, Thailand	12 weeks Groups: NDV (ND control) NDPE (ND control + prebiotic) NDPO (ND control + probiotic) NDC (ND control + synbiotic) HFV (HFD diabetic control) HFPE (HFD + prebiotic) HFPO (HFD + probiotic) HFC (HFD + synbiotic)	*Lactobacillus paracasei HII01* 10^8 CFU	Fasting insulin, HOMA-IR and AUC after OGTT	In treated groups, insulin level, HOMA-IR and the AUC after OGTT decreased significantly (p < 0.05).	All supplemented groups restored gut and systemic inflammation, cognition function through gut-brain axis, with improved hippocampal plasticity and attenuated brain mitochondrial dysfunction. Hippocampal oxidative stress and apoptosis were significantly decreased, as well as microglial activation leading to restored cognitive function. Lipid parameters (total cholesterol and LDL-c) were also improved.
Morshedi et al. [38] 2018, Iran	8 weeks Groups: HC (ND control) DC (HFD diabetic control) DL (HFD diabetic + probiotic) DI (HFD diabetic + prebiotic) DLI (HFD diabetic + synbiotic) DSh (HFD diabetic sham)	*Lactobacillus plantarum ATCC 8014* 10^7 CFU	Serum insulin	Insulin level increased in the probiotic group compared to the DSh group (p = 0.013), without difference among the supplemented ones (DL, DI, DLI).	LC group had lower plasma glucose, HbA1c, leptin, TNF-α, IL-6, LDL-c and and higher HDL-c levels. Probiotic treatment increased expressions of phosphatidylinositol-3-kinase and glycogen synthase genes and decreased of glycogen synthase kinase 3 beta mRNA. Pancreas histology showed recovery of islets cells. Also, improved SCFA production and decreased *Firmicutes*-*to*-*Bacteroidete*s ratio were observed.
Wanchai et al. [36] 2018, Thailand	12 weeks Groups: ND (ND control) NDL (ND + probiotic) HF (diabetic control) HFL (HFD + probiotic)	*Lactobacillus paracasei HII01* 10^8 CFU	Fasting insulin, HOMA-IR and response to OGTT	Insulin level and HOMA-IR were reduced significantly following probiotic supplementation, as well as plasma glucose levels during OGTT (p < 0.05).	Probiotic supplementation diminished hyperlipidemia (triglycerides and LDL-c) and systemic and kidney inflammation, endoplasmic reticulum stress and apoptosis, leading to a better kidney function. This reduced LPS levels, phosphorylation of NF-kB and c-Jun N-terminal kinase and expressions of TNF-α, IL-6, IL-1B and MCP-1 genes and ameliorated phosphoenolpyruvate carboxykinase expression.
Natividad et al. [30] 2018, France	12 weeks Groups: CD MRS (ND control) CD *L. reuteri* (ND + probiotic) HFD MRS (HFD diabetic control) HFD *L. reuteri* (HFD + probiotic)	*Lactobacillus reuteri CNCM I* *5022*-10^9 CFU	Fasting insulin, HOMA-IR, AUC after OGTT and glucose tolerance during ITT	The HFD L. reuteri group improved HOMA-IR and glucose clearance and showed a better insulin sensitivity than the non-supplemented mice.	Probiotic group improved liver steatosis, alanine aminotransferase and triglycerides concentrations, intestinal barrier function and restored GLP-1 secretion.
Yao et al. [39] 2019, China	16 weeks Groups: ND (ND control) HFD (HFD diabetic control) L (HFD + N1115) FOS (HFD + FOS) Synbiotics (HFD + FOS + L)	*Lactobacillus paracasei N1115* 2.2 × 10^9 CFU	Fasting insulin, HOMA-IR and AUC after IPGTT	In the 3 supplemented groups, insulin levels and HOMA-IR returned to baseline values and the glucose AUC after IPGTT was improved.	Alterations in inflammatory pathways (TLR4, NF-κB, p38 MAPK) and expression of tight junctions’ proteins (occludin-1 and claudin-1) were restored mainly in the synbiotic-treated animals. Treated groups showed improvements in lipid profile, serum and hepatic TNF-α, IL-1B and monoamine oxidase, postponing cirrhosis.

AUC: area under the curve; GLP-1: glucagon-like peptide-1; HDL-c: high density lipoprotein; HFD: high fat diet; HOMA: homeostasis model assessment; IL: interleukin; IPGTT: intraperitoneal glucose tolerance test; IR: insulin resistance; ITT: insulin tolerance test; IVGTT: intravenous glucose tolerance test; LDL-c: low density lipoprotein; LPS: lipopolysaccharide; MCP-1: monocyte chemoattractant protein-1; MDA: malondialdehyde; ND: normal diet; NF-κB: nuclear factor kappa B; OGTT: oral glucose tolerance test; SCFA: short-chain fatty acid; SOD: superoxide dismutase; SOD: superoxide dismutase; TNF-α: tumor necrosis factor

Except for one 4-week study [27], all probiotic interventions had significant results in at least one measure of insulin resistance. In all studies using single-strain or multiple-strains probiotics, treated arms showed improvement in the glucose metabolism.

#### Additional findings obtained with single-strain probiotics

##### Lactobacillus

In a Swedish study of HFD-fed mice supplemented with *L. plantarum*, the lack of response only to intravenous glucose tolerance test was attributed to the administration route that was unable to stimulate glucagon-like peptide-1 (GLP-1) and insulin release [18]. *L. plantarum* was also used in a Korean study after having been selected from 426 strains [7]. Reduction in insulin resistance parameters was accompanied by cellular evidence of improvement in insulin signaling and expression of genes involved in inflammation.

An intervention with *L. casei* in fructose-fed hyperinsulinemic mice increased abundances of *Lactobacillus* and *Bifidobacterium* and decreased *Clostridium*. A lower insulinemia during oral glucose tolerance test (OGTT) was associated with GLP-2 reduction mediated by *Bacteroides fragilis* [25]. Benefits in markers of liver function and gene expressions were also observed.

Administration of *L. coryniformis* to HFD-fed mice induced vascular benefits that could be related to changes in microbiota composition and intestinal permeability with reduction in metabolic endotoxemia [28].

In a study conducted in Taiwan, lyophilized and live *L. reuteri* supplementations increased *Lactobacillus* and decreased pathogens’ abundances improving intestinal barrier. Both reduced weight gain and induced improvement in lipid profile and hepatic steatosis, inflammation and gene expressions associated with insulin resistance [29]. *L. reuteri* was also used in a French study which evaluated the effects of its supplementation and an Aryl Hydrocarbon Receptor agonist, previously associated with MS [30]. The supplementation improved glucose metabolism and hepatic alanine transaminase levels.

In a Korean study, HFD-fed mice supplemented with *L. sakei* gained less weight and epididymal fat, reduced inflammation and improved the intestinal barrier (increased gene expressions of tight-junction proteins) [31].

Two studies regarding the effects of pioglitazone and different doses *L. casei* in diabetic mice showed comparable reductions in insulin resistance, TNF-α and IL-6 at 10^9^ CFU dose [16, 32]. Probiotic-induced improvement of glucose homeostasis was associated with increased abundance of *Lactobacillus* and *Bifidobacterium* and SCFA levels. Histologically, probiotic intervention prevented pancreatic islets degeneration and/or induced regeneration.

A study comparing metformin, vildagliptin and three strains of *L. rhamnosus* (GG, MTCC5690, MTCC5689) improved areas under the curve during insulin tolerance test and OGTT, except for the HFD-MTCC5690-treated [33]. Probiotics improved intestinal permeability, increased adiponectin and reduced inflammatory markers. Metabolic benefits achieved with probiotics were similar to those obtained with metformin and vildagliptin.

An Indian study in diabetic mice found better effects in glucose metabolism, lipid profile and markers of oxidative stress with *L. rhamnosus NCDC17* than with *L. rhamnosus GG* supplementation [34]. The former induced reduction in inflammatory markers (TNF-α and IL-6) and elevation in expressions of adiponectin, pro-glucagon and prohormone convertase genes.

*Lactobacillus rhamnosus* was administered in HFD-fed mice to reduce *Bilophila wadsworthia* abundance which is associated with MS [21]. Supplementation prevented the *B. wadsworthia* increase in parallel to the benefits in glucose metabolism, but only under condition of high abundance of these bacteria, probiotic ameliorated the consequences of HFD.

In a Japanese study that used the strain *L. gasseri* in fermented milk, the supplementation increased insulin secretion by the suppression of pancreatic and systemic inflammation [35].

In a Thai study, *L. paracasei* administered to obese rats induced benefits in glucose and lipid metabolism and in several markers of renal function and inflammation [36].

Four studies compared the effects of probiotics, prebiotics and synbiotics (combination of pro- and prebiotics). In all, *Lactobacillus* was used, and prebiotics were xylooligosaccharide (XOS), inulin or fructooligosaccharides (FOS). The first one evaluated the dysbiosis and inflammation in obese rats; the synbiotic group had better outcomes [8]. Treated groups decreased *Firmicutes*-*to*-*Bacteroidetes* ratio and LPS levels, but *Bifidobacterium* abundance increased only in the prebiotic group. Authors concluded that prebiotics, probiotics and synbiotics contributed to reduce endotoxemia and inflammation. The second examined the gut-brain axis in obese insulin-resistant rats by analyzing the relationship of cognitive function and administrations of *L. paracasei,* XOS and their combination [37]. Prebiotic, probiotic, and synbiotic interventions improved glucose and lipid metabolism, but only XOS and synbiotic attenuated adiposity. All treated groups restored cognitive function (improved hippocampal plasticity, brain mitochondrial function and decreased microglial activation). The third study investigated association between psychiatric disorders and oxidative stress after *L. plantarum* supplementation, inulin or their combination [38]. All interventions improved oxidative stress with beneficial psychotropic effects (depressive and anxiety-like behaviors). In the fourth study, in which *L. paracasei*, FOS or the combination was administered to HFD-fed mice, the effects on non-alcoholic fatty liver disease (NAFLD) were evaluated [39]. Benefits were found during intraperitoneal GTT in all treated groups as well as amelioration of intestinal barrier, reduction in LPS levels and activation of insulin signaling pathways. Steatosis, dyslipidemia and inflammation were improved.

##### Bifidobacterium

A study [40] that examined the role of sterilized *B. longum* in obese mice observed less weight gain, visceral fat accumulation and inflammation in parallel to improvement in glucose and lipid metabolism.

A comparison of *B. lactis* and *B. longum* supplementations showed better performance of the former regarding acetate levels elevation and metabolic benefits [41].

Two doses of *B. breve* had similar effects in reducing weight gain and visceral fat and in gene expressions in HFD-induced diabetic mice [19].

In an experiment with *B. animalis*, authors proposed that the probiotic could have increased beneficial commensal bacteria reducing endotoxemia and inflammation [20].

##### *Akkermansia muciniphila*

*Akkermansia muciniphila* was administered to metabolically healthy mice showed benefits in glucose metabolism in parallel to improvement in endoplasmic reticulum stress and inflammation [26].

In a study of metformin-treated diabetic rats, different doses of live and pasteurized *Akkermansia* were compared [27]. Independently of the dose, probiotic had no change in glucose tolerance, in contrast to metformin that induced a lower glycemic peak during the tests. Both *Akkermansia* preparations reduced hepatic glycogen and inflammation markers levels.

##### Clostridium

Supplementation with a butyrate-producing strain, *C. butyricum,* resulted in benefits in glucose and lipid metabolism that were accompanied a better intestinal barrier and anti-inflammatory effects [15].

#### Additional findings obtained with multiple-strains probiotics

One study used a mix of *Lactobacillus* and *Bifidobacterium* [42] and another two *Lactobacillus* strains plus one of *Bifidobacterium* [43].

In HFD-induced obese mice, *Lactobacillus* and *Bifidobacterium* mixture induced favorable changes in microbiota composition, body adiposity, insulin resistance and dyslipidemia [42]. Comparing the isolated effect of each probiotic against the mixture, more pronounced protective effect of the mixture was attributed mainly to the *B. animalis*.

In a Brazilian study, a mixture of three probiotics was able to modulate microbiota composition and improved intestinal permeability, endotoxemia and inflammation [43]. The intervention was also associated with reduction in hypothalamic insulin and leptin resistance, affecting feeding behavior (reduced food intake and weight gain).

### Clinical trials

Outcomes related to insulin sensitivity or resistance varied among the clinical trials as shown in Table 2. In five, significant improvement in at least one parameter [17, 22–24, 44] was observed while in two others no benefit in insulin sensitivity was found [40, 45]. Three studies used single strains of *Lactobacillus* [24, 44, 46], one proof-of-concept exploratory study used single strain of *Akkermansia muciniphila* [23] and other three tested multi-strains (two, three and six strains containing *Lactobacillus* and *Bifidobacterium*) [17, 22, 45].

**Table 2:** Description of the included clinical trials

References	Study design	Participants’ characteristics	Protocol and groups	Probiotic and dose	Insulin resistance parameter	Effects on glucose metabolism	Other outcomes
Rajukmar et al. [44] 2014, Japan	Randomized single-blind	45 healthy subjects, 20-25 years, BMI 18.5–24.9 kg/m^2^	Control, probiotic or synbiotic (FOS) group treated for 6 weeks	*Lactobacillus salivarus UBL S22* 4 × 10^9 CFU	Insulin, HOMA-IR	There were reductions (p < 0.05) in HOMA-IR in probiotic and synbiotic groups, greater in the latter. Insulinemia dropped in all groups (p < 0.05), mainly in the synbiotic group.	BMI decreased only in the synbiotic group (p < 0.05). Probiotic and symbiotic groups had significant increase in HDL-c and reductions in total cholesterol, LDL-c, triglycerides and inflammatory markers (hs-CRP, IL-6, IL-1b and TNF-α) with better results in symbiotic one.
Tripolt et al. [24] 2015, Austria	Randomized placebo-controlled	30 subjects with metabolic syndrome, 52 ± 11 and 55 ± 9 years	Control and probiotic treated for 12 weeks	*Lactobacillus casei shirota* 1.95 × 10^10 CFU	Insulin, HOMA-IR, ISI, Matsuda index, QUICKI	Probiotic-treated group improved ISI (p < 0.01) but insulinemia, HOMA-IR, QUICKI and Matsuda did not differ between groups.	Trimethylamine N-oxide levels reduced in both groups and did not differ between them and were not correlated to HOMA-IR.
Firouzi et al. [17] 2016, Malaysia	Randomized double-blind controlled parallel	136 type 2 diabetic subjects under glybenclamide/metformin, 30–70 years, BMI 18.5–40 kg/m^2^	Placebo or probiotics group treated for 12 weeks	*Mix of Lactobacillus acidophilus, Lactobacillus casei, Lactobacillus lactis,* *Bifidobacterium bifidum,* *Bifidobacterium longum,* *Bifidobacterium infantis* 10^10 CFU each strain	Insulin, HOMA-IR, QUICKI	Group supplemented with mix of strains improved (p < 0.05) insulin levels and HOMA-IR, while placebo group showed only a trend. QUICKI index did not change.	There was an improvement in HbA1c in the probiotic supplementation group compared to placebo. Participants with normal weight had significant improvement in HbA1c and triglycerides with probiotics supplementation when compared to Ow/Ob participants.
Soleimani et al. [22] 2017, Iran	Randomized double-blind placebo-controlled parallel	60 type 2 diabetic subjects under hemodialysis	Placebo or probiotic groups treated for 12 weeks	*Mix of Lactobacillus acidophilus, Lactobacillus casei, Bifidobacterium bifidum* 2 × 10^9 CFU of each strain	Insulin, HOMA-IR, QUICKI	Group supplemented with a mix of strains had reductions in insulin levels and HOMA-IR (p < 0.05), and an increase in the QUICKI index, indicating improvement in insulin sensitivity.	Subjects who received probiotic supplements showed benefits on biomarkers of inflammation and oxidative stress (hs-CRP, MDA and total antioxidant capacity). They had significant decreases in HOMA-beta, HbA1c, subjective global assessment scores and total iron binding capacity.
Tonucci et al. [45] 2017, Brazil	Randomized double-blind placebo-controlled	50 type 2 diabetic subjects	Control and probiotic treated for 6 weeks	*Mix of Lactobacillus acidophilus La*-*5, Bifidobacterium animalis BB*-*12* 10^9 CFU of each strain	Insulin, HOMA-IR	There was no significant change in insulin levels and HOMA-IR in the groups.	Treated group showed significant decreases in fructosamine, HbA1c, total cholesterol and LDL-c levels. Acetate production increased (p < 0.01) and decreased inflammatory status (TNF-α and resistin levels) in both groups. IL-10 reduced (p < 0.001) only in the control group.
Hsieh et al. [46] 2018, Taiwan	Randomized double-blind placebo-controlled	68 type 2 diabetic subjects, 25–70 years, BMI > 18.5 kg/m^2^	3 groups: placebo, live *L. reuteri* ADR-1 or heat killed *L. reuteri* ADR-3 treated for 24 weeks	*Lactobacillus reuteri ADR*-*1* 4 × 10^9 CFU *or* *Lactobacillus reuteri ADR*-*3* 2 × 10^10 CFU	Insulin, HOMA-IR	Supplemented groups had no significant difference in insulin and HOMA-IR.	Live *L. reuteri* ADR-1 treated group showed reduction in HbA1c and cholesterol, while heat-killed *L. reuteri* ADR-3 group decreased blood pressure and the inflammatory cytokine IL-1β. ADR-1 group decreased levels of aspartate aminotransferase, alanine aminotransferase and antioxidant proteins (GPX and SOD).
Depommier et al. [23] 2019, Belgium	Randomized double-blind placebo-controlled pilot study	32 insulin resistant, overweight/obese subjects, 18-70 years, BMI > 25 kg/m^2^	3 groups: placebo, pasteurized *A. muciniphila,* live *A. muciniphila treated* for 12 weeks	Pasteurized *A. muciniphila* 10^10 CFU *A. muciniphila live* 10^10 CFU	Insulin, HOMA-IR	Participants receiving both preparations of *A. muciniphila* reduced insulin levels in ~30%; this effect was significant between the pasteurized *A. muciniphila* and placebo groups. Both improved HOMA-IR.	Supplementations were safe. Pasteurized *A. muciniphila* decreased LPS and dipeptidyl peptidase IV activity. This preparation reduced white blood cells count, total cholesterol, LDL-c, AST but not ALT. Whole-body tissue damage and muscle-specific injury were attenuated as reflected by decreased lactate dehydrogenase and creatine kinase levels.

FOS: fructooligosaccharides; GPX: glutathione peroxidase; HbA1c: glycated hemoglobin; HDL: high density lipoprotein; HOMA: homeostasis model assessment; Hs-CRP: high-sensitivity C-reactive protein; IL: interleukin; IR: insulin resistance; ISI: insulin sensitivity index; OGTT: oral glucose tolerance test; Ow/Ob (overweight/obese); QUICKI: quantitative insulin sensitivity check index; LDL-c: low density lipoprotein; SOD: superoxide dismutase; TNF-α: tumor necrosis factor

#### Additional findings obtained with single-strain probiotic interventions

Four single-strain interventions were found: one in healthy subjects [44], two in those with MS [23, 24] and in one with type 2 diabetes [46].

In 45 Japanese young subjects [44], a 6-week supplementation with *L. salivarius* UBL S22, combined or not with prebiotic FOS, improved inflammatory markers and lipid profile significantly in parallel to the improvement in insulin resistance. These effects were accompanied by increase in *Lactobacillus* abundance and decrease in reactive oxygen species production in intestinal mucosa. Performance of the synbiotic group was better than the probiotic group.

An Austrian study [24] evaluated the impact of *L. casei Shirota* supplementation for 12 weeks on the levels of trimethylamine N-oxide (TMAO) in 30 subjects with MS. Insulin sensitivity index improved but not other parameters such as HOMA-IR, quantitative insulin sensitivity check index (QUICKI) and Matsuda. No benefits were found in lipid or inflammation markers. TMAO levels decreased significantly in both groups and did not differ between them.

A Taiwanese placebo-controlled clinical trial of 68 type 2 DM subjects treated with a single strain of *L. reuteri* ADR-1 or ADR-3 [46], no change in insulin sensitivity was observed, but the *L. reuteri* ADR-1-treated group showed reductions in glycated hemoglobin (HbA1c), cholesterol, and antioxidants proteins (GPX and SOD), while the ADR-3-treated group had blood pressure and IL-1β reduced.

A small clinical trial with *A. muciniphila,* published as proof-of-concept [23], showed that live or pasteurized probiotics in high doses were safe and well tolerated. Treatments restored gut microbiota and barrier function and improved several metabolic parameters. Both preparations were effective but pasteurized had a better performance.

#### Additional findings obtained with multiple-strains probiotic interventions

In a Brazilian placebo-controlled study, individuals under antidiabetic treatment who consumed fermented goat milk containing *L. acidophilus* and *B. animalis* did not change insulin resistance parameters but HbA1c, lipids and inflammatory markers were improved [45].

In a Malaysian placebo-controlled trial of diabetic individuals, HOMA-IR and HbA1c was improved by multiple-strains of probiotic intervention [17].

In an Iranian controlled study, diabetic individuals on hemodialysis treated with multiple strains of *Lactobacillus* also had HbA1c reduction in addition to improvement in insulin sensitivity indexes [22].

## Discussion

This systematic review is the first to focus on the role of probiotics in attenuating insulin resistance, involved in prevalent diseases of the contemporary world. Body of evidence was mostly based on experiments conducted in animals that supported benefits of certain supplements in attenuating induced states of insulin resistance. Underlying mechanisms and markers of improvement in insulin signaling were consistently demonstrated. In humans, existing literature are limited, and heterogeneity of study designs and samples allowed collecting reliable findings but impeded meta-analysis. Actually, most studies have been published in the last 5 years, motivated by the growing knowledge on the importance of the gut microbiome for cardiometabolic health. Despite promising results as an adjuvant therapeutic modality for controlling disorders linked by insulin resistance, there is still insufficient data in humans to support widespread probiotic prescription.

### Probiotic, its dose and duration of interventions

Most used probiotics in research and clinical settings were species of *Lactobacillus* whose benefits for health have been demonstrated [5, 44, 47]. These commensal gram-positive bacteria belong to phylum *Firmicutes*. Beneficial effects to the gastrointestinal tract, immune system, metabolism and anticarcinogenic properties have been reported [4, 5, 9]. *Bifidobacterium* (phylum *Actinobacteria*) are anaerobic and, as *Lactobacillu*s, have been added to fermented products. Optimal doses of these probiotics were already known [4, 9] and even sterilized bacteria have been considered functional [27, 29, 40]. Certain butyrate-producing species of *Clostridium* (phylum *Firmicutes*) have been associated with metabolic benefits, as well as the *A. muciniphila*, a mucin-degrading bacterium (phylum *Verrucomicrobia*). In contrast to the other probiotics, literature about *Akkermansia* is not abundant; safety and tolerability of live and pasteurized *A. muciniphila* were already investigated [23]. Researchers have used *A. muciniphila* as a pasteurized probiotic [27, 29, 40] because the pasteurization process potentializes its effects in the host [23, 48].

Some authors compared effects of probiotics and synbiotics in animals and both strategies resulted in benefits to intestinal barrier function and restored immune response [15]. This is partially in agreement with a trial in which a better performance was detected for the synbiotic group [44]. Explanation was based on the fact that acetate and lactate produced by *Lactobacillus* and *Bifidobacterium* can be converted to butyrate by other intestinal bacteria.

Most studies worked with probiotic doses from 10^8^ to 10^10^ CFU/day in both human and animal. It is necessary to maintain a content higher than 10^6^ CFU in the intestine to play an appropriate probiotic role [16]. In all studies reviewed except one [27], any dose in that range administered resulted in significant improvement in insulin resistance [16, 40] and doses higher than 10^10^ had no additional benefit [16]. Regarding live *A. muciniphila*, experiments in animals suggested that it may be required low doses and an interval between them since, in excess, could exacerbate its mucolytic function and can be harmful to the intestinal mucosa [27]. However, preliminary results obtained in a recent clinical trial, a 10^10^ CFU dose showed to be well tolerated and safe [23].

The duration of probiotic administration seemed crucial for the intervention efficacy. 12-week compared to 6-week interventions showed better results especially in animals but also in humans. Interestingly, in human studies, benefits in certain parameters of glucose metabolism and inflammatory markers were found although improved insulin sensitivity was not confirmed. We speculate that 12 week-intervention may be insufficient to get an impact on insulin sensitivity and longer trials should be further conducted. Also unknown is how persistent is the effect of the probiotic therapy when it ceased. Possibly, the probiotic-induced changes in microbiota are not permanent and its composition will return to be driven by dietary habits. Even fecal transplantation that has shown promising results in humans with obesity and SM cannot be considered as a definitive therapy for insulin resistance [9, 49].

### Probiotics and insulin resistance in animal studies

All animal studies probiotic interventions reviewed had control groups and their results were appropriately compared. The vast majority used HFD as a strategy for inducing weight gain, insulin resistance and impaired glucose tolerance, and rarely fructose or streptozotocin. This has been considered an adequate model to mimic the impact of Western diet in humans. There is consistent evidence of gut microbiota changes caused by high saturated fat intake, but controversial findings related to *Firmicutes/Bacteroidetes* ratio were reported, with increased [8], neutral [41] and even decreased [43] results among the studies. This dysbiosis elevates LPS content and compromises intestinal mucosal integrity. Locally, LPS reduces expression of tight junctions’ proteins impairing the barrier function. LPS translocation from the lumen into circulation can trigger metabolic endotoxemia; its binding to TLR-4 activates transcription of NF-κB and production of inflammatory cytokines that deteriorate insulin signaling. Therefore, a state of systemic low-grade inflammation and insulin resistance may be resultant from an inadequate diet. In the articles reviewed, HFD or fructose-enriched diet were effective to induce weight gain, systemic inflammation and a variety of metabolic abnormalities.

As the main outcome of the present review was insulin resistance or insulin sensitivity at least one parameter was reported. All except one animal study used indirect methods to assess insulin sensitivity. The gold standard method, euglycemic hyperinsulinemic clamp, was employed only in animal models in a Brazilian study by Baggaroli et al. [43]. Other investigators used indirect methods: HOMA-IR [7, 8, 16, 19, 21, 28–30, 32, 33, 36, 37, 39, 42], areas under the curve obtained by OGTT [7, 8, 15, 16, 18, 20, 21, 25, 27–37, 40, 41], intravenous [18] and intraperitoneal GTT [20, 26, 39, 42, 43], ITT [7, 15, 30, 32, 33, 40], which are reliable to estimate insulin sensitivity and contributed for interpretations of the probiotics’ effects.

Several studies, based on diverse probiotic preparations, reported their findings in different organs, also at molecular level and changes in gene expressions [16, 19, 25, 29, 32–34, 36, 38, 47] involved in underlying mechanisms of oxidative stress, inflammation and insulin resistance. HFD-induced mice showed worsening in proinflammatory cytokines and claudin and occludin gene expressions in the intestinal wall which ameliorated after probiotics interventions [33, 39]. A study [15] included in the present review observed that HFD-fed mice treated with *C. butyricum* increased tight-junction proteins’ expression in parallel to LPS levels reduction. Additionally, intervention with this *Clostridium* increased SCFA, improved intestinal permeability, insulin resistance and hepatic steatosis [15].

As a matter of fact, local and systemic benefits were described in association with SCFA produced by specific bacteria [5, 6, 50]. It is known that HFD-fed obese as well as diabetic mice have reduced SCFA levels and that fermented foods containing probiotics have the ability to increase these metabolites in the colon [32]. Butyrate exerts important roles in the intestinal immune system [6, 50] and in inhibition of the NF-kB in colonocytes [15], favoring an anti-inflammatory condition. Acetate promotes GLP-1 secretion by L cells, modulating glucose metabolism, central regulation of appetite and reducing adipocytes hypertrophy [41, 51]. Studies which employed SCFA-producing bacteria as *Clostridium* [15], *Akkermansia* [26, 27] and their metabolites, may have contributed to improvements of insulin resistance parameters.

Benefits of metabolic profile from probiotics consumption by rodents supports that they occurred at least in part via changes in the microbiota composition and intestinal barrier with systemic repercussions. Such effects were described in 96.3% of the animal studies with species of *Lactobacillus* [7, 8, 16, 18, 21, 25, 28–39, 42, 43] and *Bifidobacterium* [19, 20, 40–43], the *C. butyricum* [15] and *A. muciniphila* [26] while the supplementation lasted. Considering that their beneficial effects are desirable to control cardiometabolic diseases, deepen knowledge about long-term interventions and its outcomes is needed. Animal models studied were insulin-resistant which confers risk to atherosclerosis. A recent atherosclerosis marker, TMAO [24], is dependent on choline and carnitine metabolism by intestinal bacteria action. Probiotics have been proposed as a new therapeutic target against cardiovascular disease [28, 52]. In fact, diabetic mice treated with *L. plantarum* or prebiotic FOS reverted dysbiosis, attenuated inflammatory proteins’ expression and reduction of cardiovascular risk [52]. However, *L. casei Shirota* supplementation in humans showed no difference in TMAO levels when compared to untreated subjects although contrasting findings were found in animal studies [24].

Different research protocols among the reviewed studies impeded comparisons of probiotic efficacy among them and none investigated whether there would be a residual effect after suspending probiotic administration. Anyhow, these promising results of animal studies have motivated studies in humans considering that prevalent lifestyle-related diseases are triggered by insulin resistance.

### Probiotics and insulin resistance in clinical trials

There is mounting evidence pointing out to the role of gut dysbiosis in the pathogenesis of metabolic disorders, such as obesity, type 2 DM, MS and NAFLD [47]. Since studies demonstrated that reduction in gut microbiome diversity and richness in obese subjects were associated with high risk for insulin resistance-linked diseases [47], this ecosystem emerged as a target to improve lifestyle-related metabolic disturbances [53].

Our review highlights the scarcity and heterogeneity of human studies which tested the efficacy of probiotic supplementation in attenuating insulin resistance and reducing its cardiometabolic consequences. Sample characteristics and intervention design (probiotic type, dose and duration) differed among the studies reviewed. Four studies used single strains for 6, 12 and 24 weeks, in healthy young subjects [44] or with MS [23, 24] or type 2 DM [46], respectively, while others used multi strains blends for 6 [45] or 12 weeks [17, 22]. Also, different assessment of insulin sensitivity/resistance was employed; all trials used at least insulinemia and HOMA-IR as parameters of insulin resistance and none of the clinical trials used the gold standard technique. Investigators also used other indirect methods like insulin sensitivity index (ISI) [24], Matsuda [24] and QUICKI [17, 22, 24] to estimate the probiotics’ effects on insulin sensitivity. In the longest intervention (24 weeks) including diabetic subjects, no change in insulin resistance was observed [46], but in shorter ones [17, 22]. Subjects with MS treated with *Lactobacillus* [24] improved the ISI without changing HOMA-IR, in contrast to the intervention with *A. muciniphila* [23] that reduced HOMA-IR. In healthy subjects [44], 6 week-supplementation was sufficient to reduce insulinemia and HOMA-IR. Facing such methodological heterogeneity, no meta-analysis was available in literature.

Articles reviewed suggested probiotics use as preventive or adjuvant therapy for metabolic diseases, such as obesity and type 2 DM [44, 46]. In general, when excessive body weight was present probiotic supplementation appeared to be more effective than in eutrophic individuals [17], but conflicting findings were found among the trials [17, 22, 24, 44]. We hypothesized that normal-weight subjects on a healthy diet should already have a balanced microbiota composition, an adequate intestinal barrier and a normal immune system functioning. However, even in healthy young eutrophic subjects, a Japanese study obtained improvement in several metabolic parameters with probiotic supplementation.

The *Bacteroidetes*-*to*-*Firmicutes* ratio has been used to reflect the association of gut microbiota with body adiposity also in humans [54]. Increased ratios were reported in obese individuals or in populations consuming obesogenic diets [9], although results were not consistent [17, 46]. Weight loss in obese subjects was able to change microbiota composition without altering proportions of Bacteroidetes and Firmicutes [55]. Crosstalk between intestinal bacteria and host is relevant to determine the inflammatory state and glucose tolerance [26, 53, 56]. In fact, the role of the cytokine IL-22 in maintaining integrity of the intestinal barrier with attenuated metabolic disorders was demonstrated [57]. In the trials reviewed, such possible benefit of probiotics’ supplementation was not investigated. Association of SCFA-producing bacteria and decrease in inflammation and insulin resistance was previously reported in animals and humans [5, 6, 9]. Particularly, a role for butyrate was described in the prevention of obesity by regulating incretins and anorexigenic hormones production (GLP-1 and peptide YY), appetite and energy expenditure [47]. In our review, SCFA-producing bacteria supplemented in humans used were *A. muciniphila* and strains of *Lactobacillus and Bifidobacterium* [17, 22–24, 44–46].

Protective effects of the administration of *A. muciniphila* in intestinal barrier and metabolic disorders have been mostly investigated in animals [26, 27]. More recently, growing evidence has indicated potential benefits in individuals with obesity and type 2 DM [23, 58]. The first randomized, double-blind, placebo-controlled trial, using live or pasteurized *A. muciniphila*, in a small sample of insulin-resistant individuals, looked at safety and tolerability but also at metabolic parameters. Improvement in insulin sensitivity and other parameters have motivated several ongoing trials.

### Probiotics compared to antidiabetic agents

Interest of the scientific community has grown regarding the application of probiotics as an adjuvant therapy for insulin resistance syndromes. Currently, there are some indications of certain strains for preventive or adjuvant therapeutic purposes [4, 6, 9, 45]. Although probiotics in combination with drugs could be valuable in the treatment of prediabetes and type 2 DM, the studies reviewed analysis does not assure the efficacy of their isolated use. As insulin resistance is part of the natural history of cardiometabolic diseases, established interventions (lifestyle changes and medications) should be employed. The use of probiotics by at-risk individuals, together with behavioral measures (diet and physical activity), seemed to be useful according to preliminary results, although require confirmation in further studies.

Three animal studies that compared probiotics (*L. casei* CCFM449, *L. rhamnosus* GG, MTCC5689, MTCC5689) to antidiabetic agents (pioglitazone, vildagliptin and metformin) found similar significant improvement in insulin resistance and inflammatory markers [16, 32, 33]. In contrast, the combination of probiotics and/or prebiotics with antidiabetic drugs (metformin and sitagliptin) improved the incretin effect and insulin sensitivity in mice [59]. Although vildagliptin acts directly in GLP-1 regulation and metformin also contribute to increase its levels, similar effects were identified for probiotics suggesting that regulation of GLP-1 is a possible mechanism which favors glucose homeostasis [60, 61]. These findings support that probiotics combined with antidiabetic agents may be promising in the treatment of glucose metabolism disturbances. Further studies in humans are necessary to test this hypothesis.

The scenario disclosed by the present review shows that animal studies are still important to fill gaps on the role and mechanisms by which probiotics can enhance insulin sensitivity. Also, it has been encouraging to develop clinical trials in strata of populations, using standardized experimental conditions. Not only the animal intestinal microbiome differs from humans, but there are differences among populations worldwide [24]. In addition to lifestyle, other environmental conditions, use of antibiotics and genetic factors should influence probiotics response [4]. Advances in the knowledge on metabolic effects of probiotics, prebiotics and/or synbiotics for ameliorating microbiota composition and insulin resistance could optimize outcomes of fecal transplantation in humans with MS.

Despite the amplitude of this review, the main limitation was related to heterogeneity of samples and research protocols involving a variety of probiotics used alone or in combinations. A strength of this publication is that despite having few publications, this review collected both animal and human studies. As possible effects of supplementation with multiple strains combined are unclear, we alert that inter-strain competition and even deleterious changes in microbiota composition might occur. Standardized methods that enable the comparison of different studies and the elaboration of meta-analysis were highly warranted.

In summary, this review concluded that available data regarding the effects of certain probiotics do not guarantee sustained amelioration of insulin resistance in humans. Consistent beneficial results for intestinal barrier function, immune system and metabolism were reported in animal models, which may encourage long-term randomized clinical trials in people with obesity and at cardiometabolic risk. It is possible that supplementation with probiotics in combination with medications and/or prebiotics, combined with a healthy lifestyle, will prove useful in the current world scenario. Further research is necessary to test hypothesis raised in the present review.

## Supplementary information

**Additional file 1.** Detailed procedures for the systematic review including its search equations.

## Abbreviations

DM: Diabetes mellitus
FOS: Fructooligosaccharides
GLP: Glucagon-like peptide
GPX: Glutathione peroxidase
GTT: Glucose tolerance test
HbA1c: Glycated hemoglobin
HFD: High fat diet
HOMA-IR: Homeostasis model assessment-Insulin resistance
IL: Interleukin
ISI: Insulin sensitivity index
ITT: Insulin tolerance test
IVGTT: Intravenous glucose tolerance test
LPS: Lipopolysaccharide
MS: Metabolic syndrome
NAFLD: Non-alcoholic fatty liver disease
NF-κB: Nuclear factor kappa B
OGTT: Oral glucose tolerance test
QUICKI: Quantitative insulin sensitivity check index
SCFA: Short-chain fatty acid
SOD: Superoxide dismutase
TMAO: Trimethylamine N-oxide
TNF-α: Tumor Necrosis Factor
XOS: Xylooligosaccharides
