# Supplementary material for: Probiotics supplementation and insulin resistance: a systematic review
Source: Diabetol Metab Syndr. 2020 Nov 11;12:98. doi: 10.1186/s13098-020-00603-6 (PMC7656736; doi:10.1186/s13098-020-00603-6)
Supplement: Supplementary file 1 — Additional file 1. Detailed procedures for the systematic review including its search equations. [file 13098_2020_603_MOESM1_ESM.docx]

**Supplementary Material**

**Additional file 1:** Detailed procedures for the systematic review including its search equations.

1. ***Research methodology***

This systematic review was developed based on the PRISMA guidelines (Preferred Reporting Items for Systematic reviews and Meta-analysis), composed of a checklist of 27 items and a four-step flowchart to guide the review.

Searches were made from January 1990 until January 2020 in the scientific database PubMed, database of the National Library of Medicine, using the following descriptors Medical Subject Headings (MeSH): "Probiotics", "Lipopolysaccharides", "Gastrointestinal Microbiome", "Intestinal Mucosa", "Endotoxemia", "Fatty Acids, Volatile", "Diabetes", "Type 2", "Type II", "Prediabetic State", "Insulin Resistance", "Glucose Intolerance", "Propionibacterium", "Lactobacillus", "Saccharomyces", "Bifidobacterium". “Akkermansia” and “enterotype” were also used, which do not have MeSH descriptors, but were considered important for the data collection of this review.

The descriptors were grouped by subjects into four groups as below:

1. Probiotics
2. Lipopolysaccharides OR Gastrointestinal Microbiome OR Intestinal Mucosa OR Endotoxemia OR Fatty Acids, Volatile (this is the MeSh descriptor for Short Chain Fatty Acids – SCFA in the manuscript)
3. Diabetes Mellitus, Type 2 OR Prediabetic State OR Insulin Resistance OR Glucose Intolerance
4. Akkermansia OR Enterotype* OR Propionibacterium OR Lactobacillus OR Saccharomyces OR Bifidobacterium

The four groups were linked with "AND" to return articles with at least one item from each subgroup. In addition to the MeSh indexed search, aiming to reach all published articles of interest, another search was made called "free search", using only MeSh-matching words in attempt to find publications in which a MeSh descriptor could have not been used. Then, the two searches were added using “OR” between them; repeated articles were excluded, resulting in 56 articles for screening. The screening process is shown in the flowchart depicted in Figure 1.

1. **Detailed searches in Pubmed**

- ***Free Search***

(("Probiotics") AND ("Lipopolysaccharides" OR "Gastrointestinal Microbiome" OR "Intestinal Mucosa" OR "Endotoxemia" OR "Fatty Acids, Volatile") AND (("Diabetes” AND (“Type 2" OR “Type II”)) OR "Prediabetic State" OR "Insulin Resistance" OR "Glucose Intolerance") AND (akkermansia OR enterotype* OR "Propionibacterium" OR "Lactobacillus" OR "Saccharomyces" OR "Bifidobacterium"))

- ***Indexed Search***

((("Probiotics"[Mesh]) AND ((((("Lipopolysaccharides"[Mesh]) OR "Gastrointestinal Microbiome"[Mesh]) OR "Intestinal Mucosa"[Mesh]) OR "Endotoxemia"[Mesh]) OR "Fatty Acids, Volatile"[Mesh])) AND (((("Diabetes Mellitus, Type 2"[Mesh]) OR "Prediabetic State"[Mesh]) OR "Insulin Resistance"[Mesh]) OR "Glucose Intolerance"[Mesh])) AND (((akkermansia OR enterotype*)) OR (((("Propionibacterium"[Mesh]) OR "Lactobacillus"[Mesh]) OR "Saccharomyces"[Mesh]) OR "Bifidobacterium"[Mesh])))

- **Combination of free search and indexed search** (linked by an “OR” to sum results):

(((("Probiotics") AND ("Lipopolysaccharides" OR "Gastrointestinal Microbiome" OR "Intestinal Mucosa" OR "Endotoxemia" OR "Fatty Acids, Volatile") AND (("Diabetes” AND (“Type 2" OR “Type II”)) OR "Prediabetic State" OR "Insulin Resistance" OR "Glucose Intolerance") AND (akkermansia OR enterotype* OR "Propionibacterium" OR "Lactobacillus" OR "Saccharomyces" OR "Bifidobacterium")))) **OR** (((("Probiotics"[Mesh]) AND ((((("Lipopolysaccharides"[Mesh]) OR "Gastrointestinal Microbiome"[Mesh]) OR "Intestinal Mucosa"[Mesh]) OR "Endotoxemia"[Mesh]) OR "Fatty Acids, Volatile"[Mesh])) AND (((("Diabetes Mellitus, Type 2"[Mesh]) OR "Prediabetic State"[Mesh]) OR "Insulin Resistance"[Mesh]) OR "Glucose Intolerance"[Mesh])) AND (((akkermansia OR enterotype*)) OR (((("Propionibacterium"[Mesh]) OR "Lactobacillus"[Mesh]) OR "Saccharomyces"[Mesh]) OR "Bifidobacterium"[Mesh]))))
